# Breaking the deadlock of metallo-β-lactamases: evidence and prospects for Aztreonam/Avibactam

**DOI:** 10.1080/22221751.2026.2732702

**Published:** 2026-09-22

**Authors:** Junxin Zhou, Rui Weng, Yining Ye, Xinhong Han, Huangdu Hu, Wenzeng Xu, Qiucheng Shi, Yinping Wang, Weiyi Huang, Jingjing Quan, Ping Zhang, Ying Fu, Jintao He, Zhihui Zhou, Xiaoxing Du, Dongdong Zhao, Yan Jiang, Yunsong Yu

**Affiliations:** a Department of Infectious Diseases, Sir Run Run Shaw Hospital, Zhejiang University School of Medicine, Hangzhou, People’s Republic of China; b Zhejiang Provincial Engineering Research Center of Innovative Instruments for Precise Pathogen Detection, Hangzhou, People’s Republic of China; c Department of Pulmonary and Critical Care Medicine, Regional Medical Center for National Institute of Respiratory Diseases, Sir Run Run Shaw Hospital, School of Medicine, Zhejiang University, Hangzhou, People’s Republic of China; d Department of Clinical Laboratory, Zhejiang Cancer Hospital, Hangzhou, People’s Republic of China; e Department of Infectious Diseases, Centre for General Practice Medicine, Zhejiang Provincial People's Hospital, Hangzhou, People’s Republic of China; f Department of Laboratory Medicine, The First Affiliated Hospital, Zhejiang University School of Medicine, Hangzhou, People’s Republic of China; g Department of Clinical Laboratory, Sir Run Run Shaw Hospital, Zhejiang University School of Medicine, Hangzhou, People’s Republic of China; h Centre of Laboratory Medicine, Zhejiang Provincial People's Hospital, Hangzhou, People’s Republic of China

**Keywords:** MBLs, BLBLIs, Aztreonam/avibactam, PK/PD, PBP3 insertion

## Abstract

Multidrug-resistant Gram-negative bacteria pose a significant threat to global public health, with resistance mediated by metallo-β-lactamases being particularly prominent. Aztreonam/avibactam (AZA), a novel β-lactam/β-lactamase inhibitor combination, had been approved in China and the European Union for treating complicated intra-abdominal infections and hospital-acquired pneumonia (including ventilator-associated pneumonia) in adults. The combination exhibits broad-spectrum antimicrobial activity against carbapenemase-producing *Enterobacterales*. It inhibits KPC and OXA-48, and importantly, aztreonam (ATM) is stable against metallo-β-lactamases. Current in vitro activity studies strongly support the use of AZA against MBL-producing *Enterobacterales*, while its activity against non-fermenting bacteria appears to vary depending on the species and isolate, requiring further clinical confirmation. As a prime example of “repurposing existing drugs,” its pharmacokinetics and pharmacodynamics have been thoroughly studied, and phase III studies have provided supportive safety and efficacy data, but the clinical evidence specifically concerning microbiologically confirmed MBL-producing infections remains limited and exploratory because of very small subgroup sizes. It is important to note that the combination of ceftazidime/avibactam (CZA) with ATM differs significantly from AZA in pharmacokinetic properties. Therefore, CZA plus ATM (CZA + ATM) cannot be considered an equivalent substitute for AZA. Currently, the resistance rate for AZA is low, with most cases being isolated events. The primary mechanisms of resistance involve mutations in the PBP3 gene, mutations and overexpression of β-lactamases, reduced outer membrane permeability, and overexpression of efflux pumps. Available data suggest that AZA is an important treatment option for severe infections caused by multidrug-resistant *Enterobacterales*, including MBL-producing strains, although pharmacoeconomic conclusions remain context-dependent.

## Introduction

Since the advent of antimicrobial agents, antimicrobial resistance (AMR) has posed a global public health challenge. Between 1990 and 2019, deaths directly attributable to AMR increased from 1.06 million to 1.20 million, with projections reaching 1.91 million by 2050 [1]. Carbapenem resistance has risen most rapidly, with deaths increasing by 70% from 1990 to 2021 [1]. Carbapenem-resistant *Klebsiella pneumoniae* (CRKP) is a major contributor to this mortality. In 2024, the World Health Organization continued to list carbapenem-resistant *Enterobacterales* (CRE) as a serious threat [2, 3]. In China, imipenem resistance among *Enterobacterales* rose from 3.1% in 2005–11.1% in 2024, remaining above 10% for seven years. Carbapenem-resistant *Escherichia coli* (CREC) and CRKP account for 80–90% of CRE [4, 5]. The primary resistance mechanism is carbapenemases production, with species and geographic variation. Globally, KPC is the most common in CRKP (e.g. 94% CRKP strains carried *bla*_KPC-2_ in China), while NDM predominates in CREC, especially the NDM-5 subtype [6, 7].

Recent novel β-lactam/β-lactamase inhibitor combinations (BLBLIs) are facing rising resistance. In China, the proportion of CRKP isolates resistant to CZA increased from 6.9% in 2021 to 7.6% in 2024. CZA resistance mainly arises from KPC mutations and metallo β-lactamases (MBLs), with the MBLs conferring broader resistance (including to carbapenems and most novel BLBLIs)[8]. The proportion of CRKP co-producing KPC and NDM (KN-CRKP) has risen globally from 0.03% in 2014 to 3.10% in 2023, often causing local outbreaks [9].

Certain populations show a significantly higher MBL carriage among CRKP: 5–10% in routine intensive care units, 24% in bloodstream infections among hematological malignancy patients, 16.4% in their intestinal colonizers, and as high as 60–70% in pediatric patients [10–13.] In China, other *Enterobacterales* (e.g. *Enterobacter cloacae complex* and *Citrobacter species*) also exhibit high MBL carriage rates in China [14].

Aztreonam (ATM) is the only clinically available monobactam. However, ATM alone is generally ineffective against MBL-producing CRE [15]. Biochemical studies suggest that ATM is spared from MBL-mediated hydrolysis because it binds MBLs in a nonproductive orientation: its sulfonate group interacts with the zinc centre, while the β-lactam ring is positioned away from the zinc-activated nucleophile required for hydrolysis [16, 17]. In contrast, serine enzymes readily hydrolyze ATM, rendering it inactive. Although MBLs do not hydrolyze ATM, most MBL-producing strains co-carry other serine β-lactamases, such as extended-spectrum β-lactamases (ESBLs), AmpC, or even serine carbapenemases like KPC. Avibactam is a non-β-lactam β-lactamase inhibitor that reversibly inhibits serine β-lactamases (ESBLs, AmpC, KPC, OXA-48) and is not hydrolyzed by MBLs. When combined, ATM is protected by avibactam from hydrolysis by co-produced serine enzymes, while the MBLs remain unaffected but cannot destroy ATM. This combination has been validated in animal models to overcome MBL-mediated resistance, making it the current theoretical optimal regimen for otherwise untreatable MBL-producing CRE infections [18]. This review aims to systematically summarize the pharmacodynamics, pharmacokinetics, clinical efficacy, safety profile, and resistance mechanisms of aztreonam/avibactam (AZA), thereby providing guidance for its rational clinical application.

## Literature search strategy

A literature search was conducted in PubMed for studies published from January 2010 to December 2025. The main search terms included “aztreonam-avibactam,” “aztreonam/avibactam,” “ATM-AVI,” “metallo-β-lactamase,” “MBL,” “carbapenem-resistant Enterobacterales,” “CRE,” “minimum inhibitory concentration,” “MIC,” “pharmacokinetics,” “pharmacodynamics,” “clinical trial,” and “ceftazidime-avibactam plus aztreonam.” Additional relevant articles were identified from drug instructions and regulatory documents. Eligible studies included in vitro susceptibility studies, surveillance studies, PK/PD analyses, clinical trials, observational studies, case series, safety studies, and pharmacoeconomic analyses related to AZA or CZA plus aztreonam.

## Development strategy for AZA

MBLs are classified into B1, B2, and B3 based on zinc-coordinating ligands and the number of metal ions in the active site: B1 and B3 typically contain two zinc ions, while B2 contains one [19]. Zinc ions are central to MBL function because they participate directly in substrate binding and β-lactam hydrolysis, including activation of the nucleophilic water/hydroxide required for ring opening [17]. However, sequence similarity is very low across subclasses (<20%), and even within B1 it is relatively low (∼30%), with few highly conserved residues beyond the zinc ligands [19]. These inherent characteristics fundamentally increase the difficulty of designing broad-spectrum inhibitors. At the same time, MBLs share structural similarities with certain human enzymes; inhibitors must effectively bind to the zinc ion in the active site of MBLs while avoiding inhibition of other metalloproteases or cysteine proteases in the human body to prevent off-target toxicity [20]. The importance of zinc extends beyond catalysis. During infection, host nutritional immunity restricts zinc availability, which can reduce MBL activity and destabilize non-metallated apo-MBLs in the periplasm [21]. Furthermore, in a zinc-limited infection microenvironment, bacteria can evolve MBLs with enhanced apo-form stability and higher zinc-binding affinity, thereby diminishing the efficacy of inhibitors [22]. Clinically successful enzymes such as NDM-1 have evolved strategies to overcome this pressure, including membrane anchoring, improved in-cell stability, and variants with enhanced zinc affinity or mono-zinc activity [23, 24]. Thus, zinc availability, zinc binding, enzyme stability, and catalytic activity are tightly linked, collectively representing major challenges in MBL inhibitor research and development.

Given the current critical situation regarding MBL-producing strains and the ongoing development of MBL inhibitors, therapeutic options for treating infections caused by MBL-producing strains remain severely limited. Studies testing the *in vitro* activity of ATM combined with avibactam were conducted as early as 2011, demonstrating potent bactericidal activity against CRE (including those producing MBLs). However, the commercialized AZA was not approved by the European Union until April 2024 for the treatment of patients with complicated intra-abdominal infections (cIAIs), hospital-acquired pneumonia (HAP), and complicated urinary tract infections (cUTIs). CZA received FDA approval for use in 2015. Therefore, during periods when AZA was unavailable, CZA + ATM became an alternative treatment option.

In 2017, it was first reported that combining CZA + ATM effectively overcame resistance of *Enterobacteriaceae* producing MBLs[25]. This regimen is based on the stability of ATM against MBLs, and the protection that CZA provides against hydrolysis of ESBLs and AmpC. Furthermore, the combination of the two drugs may produce a synergistic effect by simultaneously inhibiting multiple penicillin-binding proteins.

## *In vitro* antimicrobial activity of AZA

From 2011 to December 2025, 51 studies have confirmed the efficacy of AZA against *Enterobacterales*, with an overall 50% minimum inhibitory concentration (MIC_50_) value of ≤0.03–0.12 mg/L and a 90% minimum inhibitory concentration (MIC_90_) value of 0.12–2 mg/L. For CRE strains, MIC_50_ and MIC_90_ values ranged from 0.12–1 mg/L and 0.5–4 mg/L, respectively (Table 1). Notably, AZA exhibits favourable *in vitro* activity against CRE strains producing various carbapenemases, particularly against those producing MBLs. Specifically, for KPC-producing CRE, the MIC_50_ ranged from 0.12 to 0.5 mg/L, with MIC_90_ values between 0.25 and 2 mg/L; for OXA-48-like-producing CRE, the MIC_50_ ranged from 0.12 to 0.25 mg/L, MIC_90_ was 0.25–1 mg/L; for MBL-producing CRE (involving 31 studies), MIC_50_ was ≤0.03–1 mg/L, with MIC_90_ ranging from 0.25 to 1 mg/L. Furthermore, CRE strains lacking carbapenemases (such as those with porin deficiency combined with high AmpC/ESBLs production) also exhibit sensitivity, though their overall susceptibility was lower than that of carbapenemase-producing strains. Their MIC_50_ values ranged from ≤0.12–0.5 mg/L, while MIC_90_ values fell between 0.5 and 16 mg/L. It is noteworthy that two studies reported MIC_90_ values as high as 16 and 8 mg/L respectively, significantly exceeding those reported in other investigations [26, 27]. Kim et al. reported that the MIC₉₀ of AZA against non-CP-CRE strains (8 mg/L) was higher than that against CP-CRE strains (1 mg/L), though the underlying mechanism in non-CP-CRE remains unknown due to a lack of genomic analysis [27]. In contrast, Mushtaq et al. performed whole-genome sequencing on seven *E. coli* isolates with MICs ≥ 8 mg/L and identified *ftsI* insertion mutations (YRIN or YRIK after Pro333) in six strains, all of which also carried acquired AmpC (CMY-42 in five and CMY-146 in one) [26].

**Table 1.** *In vitro* activity of aztreonam/avibactam against *Enterobacterales*.

PubMed PMID	Region	Collection time	Strains	Total	Carbapenem-resistant strains	KPC strains	MBL strains	OXA-48-like strains	None-carbapenemase-producing strains
24342639	China	2011-2012	*Enterobacteriaceae*	MIC_50_/MIC_90_, ≤0.06/0.12 (n = 291)	/	/	/	/	/
30249690	INFORM Global	2012-2015	*Enterobacteriaceae*	MIC_50_/MIC_90_, 0.06/0.12 (n = 45872)	/	/	/	MIC_50_/MIC_90_, 0.25/0.5 (n = 265)	/
25963984	Global	2012-2013	*Enterobacterales*	MIC_50_/MIC_90_, 0.06/0.12 (n = 23,516)	MIC_50_/MIC_90,_ 0.25/1 (n = 577)	/	MIC_50_/MIC_90_, 0.12/1 (n = 91)	/	/
27161636	Global	2012-2014	*Enterobacteriaceae*	MIC_50_/MIC_90_, 0.03/0.12 (n = 38266)	/	MIC_50_/MIC_90_, 0.25/0.5 (n = 557)	/	/	/
28630192	Global	2012-2015	*Enterobacteriaceae*	MIC_50/_MIC_90_, 0.06/0.12 (n = 51,352)	MIC_50_/MIC_90_, 0.25/1 (n = 1498)	/	MIC_50_/MIC_90_, 0.12/1 (n = 267)	/	/
39672348	China	2014-2020	NDM-*Enterobacterales*	/	/	/	MIC_50_/MIC_90_, 0.12/1 (n = 204)	/	/
38154750	ATLAS Global	2016-2020	*Enterobacterales*	MIC_50_/MIC_90_, 0.06/0.25 (n = 82,775)	/	/	MIC_50_/MIC_90_, 0.12/1 (n = 1628)	/	/
37998793	ATLAS Global	2016-2020	*Enterobacterales*	MIC_90_, 0.12-0.25 (n = 116,602)	MIC_90_, 1 (n = 5576)	/	MIC_90_, 1 (n = 1877)	/	/
32841721	Latin America, Africa/Middle East, Asia, and Eurasia	2016-2017	*K. pneumoniae*	/	MIC_50_/MIC_90_, 0.25/0.5 (n = 583)	/	MIC_50_/MIC_90_, 0.12/0.25 (n = 114)	/	/
29061754	America	2016	*Enterobacteriaceae*	MIC_50_/MIC_90_, ≤0.03/0.12 (n = 10451)	MIC_50_/MIC_90_, 0.12/0.5 (n = 120)	MIC_50_/MIC_90_, 0.25/0.5 (n = 102)	MIC_50_/MIC_90_, 0.12/0.5 (n = 59)	MIC_50_/MIC_90_, 0.25/0.25 (n = 57)	MIC_50_/MIC_90_, 0.25/4 (n = 12)
35510161	China	2016-2017	CRE	/	MIC_50_/MIC_90_, ≤0.12/0.5 (n = 312)	MIC_50_/MIC_90_, 0.25/0.5 (n = 147)	MIC_50_/MIC_90_, ≤0.12/0.5 (n = 118)	/	MIC_50_/MIC_90_, ≤0.12/0.5 (n = 24)
37154758	Taiwan, China	2016-2021	CPE	/	MIC_50_/MIC_90_, 0.25/1 (n = 195)	MIC_50_/MIC_90_, 0.5/1 (n = 38)	MIC_50_/MIC_90_, 0.25/1 (n = 143)	MIC_50_/MIC_90_, 0.25/1 (n = 14)	/
40365080	America	2017-2022	CRE	/	MIC_50_/MIC_90_, 0.25/1 (n = 511)	/	MIC_50_/MIC_90_, 0.25/1 (n = 50)	/	MIC_50_/MIC_90_, 0.25/4 (n = 89)
40711423	Span	2017-2024	CPE	MIC_50_/MIC_90_, 0.12/0.5 (n = 300)	/	MIC_50_/MIC_90_, 0.12/0.25 (n = 100)	MIC_50_/MIC_90_, 0.12/0.25 (n = 100)	MIC_50_/MIC_90_, 0.12/0.25 (n = 100)	/
36736925	Span	2018	MBL-E	/	/	/	MIC_50_/MIC_90_, ≤1/≤1 (n = 55)	/	/
33116675	China	2018-2019	CRE	/	MIC_50_/MIC_90_, ≤0.12/1 (n = 120)	/	/	/	/
40225828	America	2018-2020	*Enterobacterales*	MIC_50_/MIC_90_, ≤0.03/0.12 (n = 45497)	/	/	MIC_50_/MIC_90_,0.25/0.5 (n = 50)	/	/
35481835	China	2018-2019	CRE	/	MIC_50_/MIC_90_,0.25/1 (n = 1158)	MIC_50_/MIC_90_,0.5/1 (n = 569)	MIC_50_/MIC_90_,0.12/1 (n = 400)	/	/
35760303	ATLAS Global	2019	*Enterobacterales*	MIC_50_/MIC_90_, 0.03/0.25 (n = 18713)	MIC_90_, 0.5 (n = 1607)	/	MIC_50_/MIC_90_, 0.12/0.5 (n = 462)	/	/
35041100	SENTRY Global	2019-2020	*Enterobacterales*	MIC_50_/MIC_90_, ≤0.03/0.12 (n = 8786)	MIC_50_/MIC_90_, 0.25/0.5 (n = 424)	/	/	/	/
37526796	Latin America, Africa/Middle East, Asia and Eurasia	2019-2021	*Enterobacterales*	MIC_90_, 0.25 (n = 24937)	MIC_90_, 1 (n = 3289)	MIC_90_, 0.5 (n = 705)	MIC_90_, 2 (n = 1610)	MIC_90_, 0.5 (n = 831)	/
36846612	America	2019-2021	*Enterobacterales*	MIC_50_/MIC_90_, ≤0.03/0.12 (n = 27834)	MIC_50_/MIC_90_, 0.25/0.5 (n = 261)	MIC_50_/MIC_90_, 0.12/1 (n = 171)	MIC_50_/MIC_90_, 0.25/0.5 (n = 33)	MIC_50_/MIC_90_, 0.25/0.5 (n = 12)	MIC_50_/MIC_90_, 0.5/2 (n = 44)
40521716	Europe	2019-2023	*Enterobacterales*	MIC_50_/MIC_90_, ≤0.03/0.12 (n = 8074)	MIC_50_/MIC_90_, 0.25/0.5 (n = 757)	/	/	/	/
33276387	Europe, Asia, Latin America	2019	*Enterobacterales*	MIC_50_/MIC_90_, ≤0.03/0.12 (n = 8787)	MIC_50_/MIC_90_, 0.25/0.5 (n = 396)	MIC_50_/MIC_90_, 0.25/0.5 (n = 138)	MIC_50_/MIC_90_, 0.12/0.5 (n = 110)	MIC_50_/MIC_90_, 0.25/0.5 (n = 87)	MIC_50_/MIC_90_, 0.5/2 (n = 39)
34900759	China	2019	CRE	/	MIC_50_/MIC_90_, 0.5/2 (n = 374)	/	/	/	/
37184411	China	2019	*Enterobacterales*	MIC_50_/MIC_90_, ≤1/≤1 (n = 1202)	MIC_50_/MIC_90_, ≤1/4 (n = 119)	/	MIC_50_/MIC_90_, ≤1/4 (n = 43)	/	/
34970142	China	2019	CRKP	/	MIC_50_/MIC_90_, 1/4 (n = 318)	/	/	/	/
38354826	Europe, Latin America and the Asia-Pacific region	2020-2022	CRE	/	MIC_50_/MIC_90_, 0.25/0.5 (n = 1016)	MIC_50_/MIC_90_, 0.25/0.5 (n = 430)	MIC_50_/MIC_90_, 0.12/0.5 (n = 356)	MIC_50_/MIC_90_, 0.25/0.5 (n = 126)	MIC_50_/MIC_90_, 0.5/2 (n = 103)
38904361	Europe	2020	*Enterobacterales*	MIC_90_, ≤1 (n = 1909)	/	/	/	/	/
39183351	ARTEMIS Europe	2020	*K. pneumoniae*	MIC_50_/MIC_90_, 1/2 (n = 187)	MIC_50_/MIC_90_, 1/2 (n = 104)	/	/	/	/
39183351	ARTEMIS Europe	2020	*E. coli*	MIC_50_/MIC_90_, 0.25/1 (n = 93)	/	/	/	/	/
39304121	China	2021	CRE	/	MIC_50_/MIC_90_, 1/1 (n = 332)	/	/	/	/
41132366	America	2021-2023	*Enterobacterales*	MIC_90_,0.12 (n = 10574)	MIC_90_,0.5 (n = 81)	/	/	/	/
33334045	South Korea	2014Jan.-2018 Oct.	CRE	/	/	MIC_50_/MIC_90_, 0.25/1 (n = 17)	MIC_50_/MIC_90_, 0.25/1 (n = 11)	/	MIC_50_/MIC_90_, 0.5/8 (n = 46)
32387260	China	2018 Jan.-2019 Jun.	CRE	/	/	MIC_50_/MIC_90_, 0.25/2 (n = 60)	MIC_50_/MIC_90_, 0.12/2 (n = 26)	/	/
37982631	China	2018 Aug.-2022 Jun.	CRKP	/	MIC_50_/MIC_90_, 1/4 (n = 114)	/	/	/	/
34060895	America	2019 Mar.-2020 Dec.	MBL-E	/	/	/	MIC_50_/MIC_90_, 0.5/8 (n = 64)	/	/
40207055	China	2021 Jan.-2024 Jun.	CRKP	/	MIC_50_/MIC_90_, 0.12/0.25 (n = 363)	/	MIC_50_/MIC_90_, 0.12/0.25 (n = 240)	/	/
32473388	China	2016 Oct.-2017 Sept.	MBL-*Enterobacteriaceae*	/	/	/	MIC_50_/MIC_90_,<0.12/1 (n = 161)	/	/
38176458	England	2019-2020	CRE	/	/	/	/	/	MIC_50_/MIC_90_, 0.5/16 (n = 51)
36968952	Europe, Asia, Latin America	2019-2021	CRE	/	MIC_50_/MIC_90_, 0.25/0.5 (n = 1098)	MIC_50_/MIC_90_, 0.25/0.5 (n = 473)	MIC_50_/MIC_90_, 0.12/0.5 (n = 347)	MIC_50_/MIC_90_, 0.25/0.5 (n = 205)	MIC_50_/MIC_90_, 0.5/2 (n = 126)
40298548	America	2019-2023	*C. freundii*	MIC_50_/MIC_90_, 0.06/0.25 (n = 1374)	MIC_50_/MIC_90_, 0.12/0.5 (n = 12)	/	/	/	/
40298548	America	2019-2023	*E. cloacae*	MIC_50_/MIC_90_, 0.06/0.5 (n = 3732)	MIC_50_/MIC_90_, 0.25/1 (n = 52)	/	MIC_50_/MIC_90_, 0.12/0.25 (n = 12)	/	/
40499756	America	2020-2021	*Enterobacterales*	MIC_50_/MIC_90_, 0.12/0.5 (n = 157)	/	MIC_50_/MIC_90_, 0.12/0.5 (n = 115)	MIC_50_/MIC_90_, 0.25/0.5 (n = 30)	/	/
38927230	America	2020-2022	*Enterobacterales*	MIC_50_/MIC_90_, 0.25/1 (n = 210)	/	/	/	/	/
39856702	America	2020-2022	*Enterobacterales*	MIC_50_/MIC_90_, 0.06/0.25 (n = 3911)	MIC_50_/MIC_90_, 0.25/1 (n = 104)	/	/	/	/
39501203	America	2020-2022	*Enterobacterales*	MIC_50_/MIC_90_, ≤0.03/0.12 (n = 4802)	MIC_50_/MIC_90_, 0.25/1 (n = 49)	/	MIC_50_/MIC_90_, ≤0.03/0.5 (n = 8)	/	/
40108737	France	/	*Enterobacterales*	MIC_50_/MIC_90_, 0.12/0.75 (n = 127)	/	/	MIC_50_/MIC_90_, 0.12/0.75 (n = 32)	/	/
40683213	France	/	CRE	/	MIC_50_/MIC_90_, 0.12/1 (n = 414)	/	MIC_50_/MIC_90_, 0.12/0.75 (n = 82)	/	MIC_50_/MIC_90_, 0.5/4 (n = 122)
26392487	America, Singapore	/	*Enterobacteriaceae*	/	/	MIC_50_/MIC_90_, 0.12/0.5 (n = 108)	MIC_50_/MIC_90_, 0.12/1 (n = 55)	/	/
36308322	Switzerland	/	MBL-E	/	/	/	MIC_50_/MIC_90_, 1/16 (n = 64)	/	/

CPE: carbapenemase-producing *Enterobacterales*; MBL-E: metallo β-lactamase-producing *Enterobacterales*; /: indicates that no relevant data were reported in the literature.

*In vitro* activity studies of AZA had also focused on *Pseudomonas aeruginosa* (*P. aeruginosa*) and *Stenotrophomonas maltophilia* (*S. maltophilia*). The carbapenemases carried by the former are primarily MBLs, such as VIM, IMP, and NDM. Whereas *S. maltophilia* naturally carries two inducible β-lactamases: L1, a subclass B3 MBL, and L2, a class A serine β-lactamase. This distinction is central to the rationale for AZA activity, because aztreonam is relatively stable to L1-mediated hydrolysis, while avibactam inhibits L2. Nine existing studies indicated that AZA exhibited an overall MIC_50_ of 8–16 mg/L and an MIC_90_ of 16–>256 mg/L against *P. aeruginosa* (Table 2). One US study reported significantly lower values, with MIC_50_ and MIC_90_ of 0.25 and 1 mg/L respectively (*n* = 2130) [28]. A recent comparative study further showed that, among 38 MDR *P. aeruginosa* isolates, CZA + ATM had lower MICs than AZA in 25 isolates, suggesting greater *in vitro* activity in some strains [29]. However, this does not imply that CZA + ATM offers any clinical advantage over multidrug-resistant *Pseudomonas aeruginosa* isolates. In comparison, *S. maltophilia* showed slightly better susceptibility to AZA, with an MIC_50_ of 2–4 mg/L and an MIC_90_ of 4–8 mg/L. This data covered nearly 3,500 clinical isolates worldwide. A recent study by Kunz Coyne et al. compared AZA with CZA + ATM against 23 *S. maltophilia* complex isolates[30]. The two regimens showed comparable overall in vitro activity, with no uniform advantage of either regimen. Semi-mechanistic pharmacodynamic modelling suggested that AZA performed better in strains with dominant L2 β-lactamase expression, whereas CZA + ATM was favoured in settings of L1 or SmeABC efflux pump dominance [30]. These modelling findings highlight that population averages may not capture genotype-dependent differences in dynamic activity. However, the MICs of AZA against both *Acinetobacter baumannii* (*A. baumannii*) and anaerobic bacteria were > 32 mg/L, indicating a lack of *in vitro* activity [31, 32].

**Table 2.** In vitro activity of aztreonam/avibactam against *Pseudomonas aeruginosa* and *Stenotrophomonas maltophilia*.

Ref	Region	Collection time	Strains	Total	Carbapenem.resistant.strains	MBL strains
24342639	China	2011-2012	*P. aeruginosa*	MIC_50_/MIC_90_, 16/>64(n = 25)	/	/
28630192	Global	2012-2015	*P. aeruginosa*	MIC_50_/MIC_90_, 8/64(n = 11842)	/	MIC_50_/MIC_90_, 16/64(n = 452)
34900759	China	2019	CRPA	/	MIC_50_/MIC_90_, 16/64(n = 65)	/
38304722	Taiwan,China	2019-2021	CRPA	/	MIC_50_/MIC_90_, 16/>32(n = 110)	/
39856702	America	2020–2022	*P. aeruginosa*	MIC_50_/MIC_90_, 0.25/1(n = 2130)	/	/
39304121	China	2021	CRPA	/	MIC_50_/MIC_90_, 8/32(n = 152)	/
33086177	/	2022-2024	*P. aeruginosa*	MIC_50_/MIC_90_, 8/16(n = 30)	/	/
40108737	France	/	*P. aeruginosa*	MIC_50_/MIC_90_, 16/>256(n = 53)	/	/
36308322	Switzerland	/	MBL-*P. aeruginosa*	/	/	MIC_50_/MIC_90_, 16/>128(n = 39)
38483164	European	/	*P. aeruginosa*	MIC_90_, 32(n = 950)	/	/
32928733	SENTRY	2008-2018	*S. maltophilia*	MIC_50_/MIC_90_, 4/4(n = 47)	/	/
40084947	Japan	2012-2024	*S. maltophilia*	MIC_50_/MIC_90_, 4/8(n = 53)	/	/
33618662	China	2014-2018	*S. maltophilia*	MIC_50_/MIC_90_, 2/8(n = 76)	/	/
33086177	/	2022-2024	*S. maltophilia*	MIC_50_/MIC_90_, 2/8(n = 24)	/	/
32900683	America	2019-2023	*S. maltophilia*	MIC_50_/MIC_90_, 2/4(n = 1400)	/	/
32900683	Global	/	*S. maltophilia*	MIC_50_/MIC_90_, 4/4(n = 1839)	/	/

CRPA, carbapenem-resistant *Pseudomonas aeruginosa*; /, indicates that no relevant data are reported in the literature.

Consequently, AZA showed variable in vitro activity against *P. aeruginosa* and *S. maltophilia*, with MIC distributions that should be interpreted cautiously because clinical breakpoints for these organisms are not established. These data do not support a general conclusion of reliable clinical activity against non-fermenters, particularly *P. aeruginosa*. In contrast, AZA lacked meaningful in vitro activity against *A. baumannii* and anaerobic bacteria (MICs >32 mg/L). Overall, the spectrum of AZA is best supported for MBL-producing *Enterobacterales*, whereas its potential role against non-fermenters remains organism- and isolate-dependent and requires further clinical validation.

## Comparative *in vitro* activity of novel BLBLIs against MBL-producing strains

*In vitro* activity of AZA against MBLs-producing Gram-negative bacteria differs markedly from that of novel BLBLIs such as cefepime/taniborbactam and cefepime/zidebactam. In a study of 490 MBL-producing *Enterobacterales* from 27 countries, AZA demonstrated the most consistent and broad-spectrum activity, with all isolates being susceptible (MIC₅₀/MIC₉₀, 0.12/0.5 mg/L) regardless of the presence of other β-lactamases, porin mutations or efflux pump overexpression [33]. In the same collection, cefiderocol showed only 66.7% susceptibility by EUCAST breakpoints, whereas cefepime/taniborbactam inhibited only 36.7% of isolates when applying cefepime breakpoints [33]. In another evaluation using 190 isogenic *E. coli* strains each overproducing a single β-lactamase, cefepime/taniborbactam displayed an overall inhibition spectrum comparable to AZA (MIC₅₀/MIC₉₀, 0.25/8 mg/L versus 0.5/4 mg/L), but lacked activity against certain MBL variants such as IMP-1, VIM-89 and NDM-9 [34]. Le Terrier et al. further demonstrated that cefepime/zidebactam, cefepime/taniborbactam, and meropenem/nacubactam were the most effective combinations in *E. coli*, whereas AZA demonstrated a minor shortcoming, with reduced activity specifically observed against strains producing PER-type and CMY-type β-lactamases [35]. A shared weakness of AZA and cefepime/taniborbactam became particularly evident in NDM-producing *E. coli* strains harbouring YRIN or YRIK insertions in penicillin-binding protein 3 (PBP3). In a collection of 28 such clinical isolates co-producing CMY-42, all strains were fully resistant to both combinations, whereas cefepime/zidebactam retained very low MICs (MIC₅₀/MIC₉₀, 0.25/0.25 mg/L), its intrinsic antibacterial and PBP2-enhancing effect remaining unaffected by PBP3 alterations [36].

In *P. aeruginosa*, the antimicrobial activity spectra of BLBLIs differed substantially. AZA remained consistently active against MBLs, while cefepime/zidebactam offered broad coverage but could be affected by certain class A and C β-lactamases. Cefepime/taniborbactam showed gaps against both IMP-type MBLs and various serine β-lactamases. The narrowest spectra belonged to meropenem/nacubactam (ineffective against nearly all MBLs and OXA-23) and cefepime/enmetazobactam (deficient against β-lactamases from all four Ambler classes) [35]. Cefepime/taniborbactam MICs were readily compromised by efflux pump overexpression. When NDM-1 production coexisted with MexAB-OprM overexpression and OprD deficiency, nearly all tested BLBLIs lost activity [37]. In this setting, AZA and cefepime/zidebactam still provided the broadest coverage against mutants expressing various acquired β-lactamases [37].

In summary, among *Enterobacterales*, AZA provides the highest coverage against MBL producers. Cefepime/taniborbactam offers similarly broad inhibition but has gaps for certain MBL subtypes. Both are inferior to cefepime/zidebactam in the presence of PBP3 insertions. In *P. aeruginosa*, drug choice must be guided by the specific combination of resistance mechanisms, with AZA remaining the most reliable option against MBL producers [35, 37].

## Pharmacokinetics/Pharmacodynamics (PK/PD) of AZA

### PK/PD of aztreonam and avibactam

ATM has extremely low oral bioavailability (<1%), requiring intravenous (IV), intramuscular (IM), nebulization, or intraperitoneal (IP) administration, with regimens ranging from 0.5 g twice daily to 2 g four times daily depending on patient condition and infection severity.

Its PK follows a two-compartment open model, with a rapid distribution phase and an elimination half-life of about 2 h, a steady-state volume of distribution of 0.18 L/kg (similar to extracellular fluid volume), and a protein binding rate of around 56%. ATM penetrates widely into tissues and body fluids, including synovial fluid (83.0 mg/L after 2 h), bone (16.0 mg/L), bronchial secretions (0.5–18.7 mg/L) and cerebrospinal fluid (CSF) [38, 39]. CSF penetration is markedly enhanced in meningitis, with concentrations ranging from 0.76 to 16.6 mg/L and reaching 39–62 mg/L after multiple doses [40, 41]. It also distributes widely to abdominal organs: after a single 2 g dose in surgical patients, concentrations range from 12 to 33 µg/g in endometrium, fallopian tubes and ovaries, and reach 65.4 mg/L in liver and 9.2 mg/L in muscle [42,43]. ATM is minimally metabolized, with approximately two-thirds excreted unchanged in urine, and only 3.1%–6.9% as its primary metabolite SQ26992 [44]. Clearance correlates directly with creatinine clearance, requiring dose adjustment in renal impairment.

In terms of PD, ATM exhibits time-dependent killing. The duration during which the free drug concentration exceeds the MIC (%*ƒ*T > MIC) has been identified as a key pharmacokinetic parameter. For *Enterobacterales*, the target %*ƒ*T > MIC is 45–70% [45], whereas for *P. aeruginosa* it is 50–60% [46]. Monte Carlo simulations supported that 1 g every 8 h achieves high probability of target attainment against *Enterobacterales*, whereas 1 g every 6 h or 2 g every 8 h is needed for *P. aeruginosa*. Therefore, the dose or frequency of administration should be increased when treating or managing suspected *P. aeruginosa* infections.

Avibactam also has low oral bioavailability and must be given intravenously. Its volume of distribution is approximately 22 L (confined to extracellular space), with low plasma protein binding (around 8%) [47]. It exhibits linear PK and good tissue penetration. Concentrations in bronchial epithelial lining fluid reach approximately 30% of plasma level, and during meningeal inflammation CSF exposure can reach 38% of plasma AUC [48]. In patients with complicated intra-abdominal infections and urinary tract infections, the central volume of distribution was 32.9% and 43.4% higher, respectively, than in healthy volunteers [49].

Avibactam is minimally metabolized and has no significant inhibitory or inducing effect on cytochrome P450, and is primarily eliminated unchanged by the kidneys. Its renal clearance (about 158 mL/min) exceeds glomerular filtration rate, suggesting active tubular secretion [50]. The elimination half-life is approximately 2 h, and exposure increases approximately 3.8-fold and 7-fold in moderate and severe renal impairment, respectively, necessitating dose adjustment [48].

Regarding PD, avibactam acts by fully inhibiting β-lactamases, so the activity of AZA is determined by the PK/PD characteristics of ATM alone. The threshold concentration (C_T_) is the minimum concentration required to restore ATM activity. For *Enterobacterales*, a C_T_ of 2.5 mg/L is recommended when combined with ATM, and free avibactam must exceed t C_T_ for ≥ 50% of the dosing interval [51]. However, when combined with ceftazidime, the required concentration is only at least 0.5 mg/L [52]. Therefore, the PD target of avibactam is higher for the AZA than CZA. For *P. aeruginosa*, the optimal C_T_ is 1 mg/L [53].

### PK/PD and dose adjustments in special populations

The PK of ATM varies significantly in specific patient populations, which is a key factor in determining clinical dose adjustments. In patients with renal impairment, its clearance correlates with creatinine clearance: when creatinine clearance is below 10 mL/min, serum clearance decreases to 29 mL/min and the half-life is prolonged to approximately 6-8 h [54]. Accordingly, for patients with creatinine clearance of 30–80, 10–29, and less than 10 ml/min or anuria, the recommended dose should be adjusted to 75%, 50% and 25% of the standard dose, respectively [54]. The reduced clearance rate in elderly patients is primarily due to age-related renal impairment. In pediatric patients, PK was age-dependent: in infants less than seven days old and weighing less than 2.5 kg, the half-life was as long as 5.71 h and the clearance was only 0.94 mL/min/kg. Therefore, dosing every 12 h was recommended. In children aged one month to two years, the half-life was approximately 1.7 h and dosing every six to eight hours was recommended [55]. Critically ill patients may require higher doses due to an increased volume of distribution, reduced protein binding, and increased clearance. The pharmacokinetic variability of avibactam is primarily related to its renal clearance, with renal function being a key factor in influencing exposure [49]. Hemodialysis effectively removes ATM and avibactam, so administration should be scheduled to take place afterwards. Avibactam is not significantly metabolized by the liver, no dose adjustment was required for patients with mild to moderate hepatic impairment.

The dosage adjustment regimen for AZA in adults based on renal function is shown in Table 3. As the population PK of this drug in pediatric patients has not yet been established, it is not approved for use in pediatrics.

**Table 3.** Dosage adjustment regimens for AZA and CZA based on renal function [50, 81].

Renal Function Classification (Based on CrCl)	AZA	CZA
Mild impairment (CrCl > 50 mL/min)	Load dose: 2 g/0.67 g; maintenance dose: 1.5 g/0.5 g, q6h	2 g/0.5 g, q8h
Moderate impairment (CrCl 30–50 mL/min)	Load dose: 2 g/0.67 g Maintenance dose: 0.75 g/0.25 g, q6h	1 g/0.25 g, q8h
Severe impairment (CrCl 15–30 mL/min)	Load dose: 1.35 g/0.45 g Maintenance dose: 0.675 g/0.225 g, q8h	0.75 g/0.1875g, q12h
End-stage renal disease (CrCl ≤ 15 mL/min) and haemodialysis	Load dose: 1 g/0.33 g; maintenance dose: 0.675 g/0.225 g, q12h	0.75 g/0.1875g, q48h

Use the Cockcroft-Gault formula to estimate creatinine clearance (CrCl). q6h: once every 6 h; q8h: once every 8 h; q12h: once every 12 h; q48h: once every 48 h.

To further characterize the PK profile of AZA, a population PK analysis was conducted, including a total of 4,914 plasma samples of ATM (from 431 subjects) and 18,222 plasma samples of avibactam (from 2,635 subjects) [56]. Body weight and time-dependent creatinine clearance were key covariates influencing the clearance of both compounds. The type of infection also significantly affected clearance and volume of distribution. Exposure was lowest in patients with cIAIs, whilst exposure and median *ƒ*T > C_T_ were generally higher in patients with HAP and cUTIs compared with those with cIAIs.

The pharmacodynamic target for AZA is the simultaneous achievement of 60% *ƒ*T > MIC (8 mg/L) for ATM and 50% *ƒ*T > CT (2.5 mg/L) for avibactam. The simulation results show that, for patients with varying degrees of renal function and different types of infection, the fixed-dose combination regimen achieves a steady-state probability of pharmacodynamic target attainment (PTA) ranging from 89% to over 99% [56].

### PK/PD non-interchangeability of AZA and CZA + ATM

Where AZA is unavailable, the Infectious Diseases Society of America (IDSA) guidelines have recommended an alternative regimen of CZA 2.5 g every 8 h combined with ATM 2 g every 8 h administered as a 3-hour infusion [57]. This regimen essentially consists of a temporary combination of two separate drugs. To understand the gap between this alternative and AZA, it is necessary to clarify the advantages of AZA. AZA uses an optimized fixed 3:1 ratio (ATM 1.5 g and avibactam 0.5 g), with its loading and maintenance doses selected based on large-scale population PK modelling and phase III clinical trial validation. It can simultaneously achieve the respective PD targets of ATM and avibactam across different renal function and infection types, with a joint PTA of 89% to >99% [56]. However, CZA + ATM has a fundamental flaw—the fixed ratio of ceftazidime to avibactam was designed to protect ceftazidime. When ATM is added to the combination, the dose of avibactam is not increased accordingly, disrupting the balance between the antibiotic and the protective agent. Population PK simulation confirmed that the steady-state exposure to avibactam in the combination regimen was approximately 25% lower than that in the fixed-dose AZA combination regimen, which directly resulted in the PD target of 50% *ƒ*T > 2.5 mg/L not being reliably achieved, ultimately causing the combined PTA to fall below 85% [56]. Moreover, a therapeutic drug monitoring study in critically ill patients showed that even with prolonged (3-hour) infusion regimen of CZA with aztreonam, avibactam achieved 50% *ƒ*T > 1 mg/L (a threshold far lower than 2.5 mg/L) in only 74% of patients, and attainment of 100% ƒT > MIC fell to 35% [58].

More critically, CZA + ATM also exhibits an incompletely recognized drug interaction and higher PK variability. A healthy volunteer study (the COMBINE study) found that when co-administered with CZA, the total clearance of ATM was unexpectedly reduced by approximately 16%, leading to increased ATM exposure and a potential risk of elevated liver enzymes [59]. In real-world complex patient populations (e.g. the elderly, burn patients, septic shock), the apparent volumes of distribution of ceftazidime, avibactam and ATM are significantly increased, while clearance is reduced by about 50% compared to typical values, with estimated glomerular filtration rate being the only stable covariate [60]. This high variability makes drug exposure unpredictable with the temporary combination, undermining the reliability of its PD targets. In contrast, the AZA PK model, based on data from over 2,600 subjects, includes clear, quantified adjustments for key covariates such as renal function, resulting in a more robust exposure–response relationship.

Taken together, AZA and CZA + ATM differ substantially in formulation, exposure predictability, and PK/PD target attainment. AZA is a fixed-dose formulation that delivers aztreonam and avibactam in a synchronized and standardized manner, supporting predictable co-exposure and joint PTA across renal function strata [61]. In contrast, CZA + ATM is a co-administration strategy using two separately prepared products. Although simultaneous or Y-site infusion can improve overlap between ATM and avibactam exposure, this regimen remains more vulnerable to infusion timing, renal function, and patient-specific PK variability [61]. Therefore, CZA + ATM should be regarded as an important practical alternative when AZA is unavailable, but not as a pharmacokinetically interchangeable substitute for fixed-dose AZA.

## Mechanisms of resistance to AZA

Since 2020, there have been a series of reports on AZA resistant strains. Due to the limited antimicrobial spectrum of AZA, certain anaerobic bacteria, *Acinetobacter baumannii* and Gram-positive bacteria are naturally resistant to it. This section focuses on reviewing the mechanisms of resistance to AZA in *Enterobacterales*.

Several studies have shown that a four-amino-acid insertion (YRIN or YRIK) following proline at position 333 of the PBP3 protein is commonly observed in *Escherichia coli* strains exhibiting reduced susceptibility to AZA [26]. These strains are predominantly NDM-producing or carbapenem-resistant strains, and frequently harbour multiple β-lactamases, including CMY-type AmpC, TEM and CTX-M [62]. A global genomic analysis (*n* = 159,341) found that 2.01% of *E. coli* strains carried PBP3 insertion variants, of which 94.75% were amino acid repeats at positions 334-337 (YRIN accounted for 65.92% and YRIK for 28.83%), followed by PYRI repeats at positions 333-336 (4.16%)[63]. These variant strains were distributed across 62 countries on six continents, primarily concentrated in high-risk clones ST410, ST167 and ST405, with 83.32% carrying NDM. MBL-producing CREC strains often have insertional mutations in PBP3. This is a significant resistance mechanism, but it needs to work together with other β-lactames, like AmpC, because it is not enough on its own to cause resistance.

This mode of synergy is congruent with the resistance mechanisms of CZA, wherein β-lactamase mutations and overexpression play a significant role in resistance to AZA. Ma et al. reported that the presence of both KPC-21 (a Trp105Arg mutation in KPC-2) and a YRIN insertion in PBP3 led to high-level resistance to AZA (MIC = 512/4 mg/L) in *E. coli*, while susceptibility to CZA remained [64]. Cloning experiments demonstrated that KPC-21 alone conferred only a slight increase in MIC, but significantly enhanced resistance in combination with the PBP3 insertion. Xiang et al. identified a KPC-2 Ser109Pro mutation in CRKP that reduced affinity for avibactam and ATM [65]. Ser109Pro significantly increased resistance in the presence of an OmpK36 deletion and high-level KPC expression.

When PBP3 harbours a YRIN or YRIK insertion, certain CTX-M-type ESBLs capable of efficiently hydrolyzing ATM can also mediate resistance. For example, CTX-M-15 and its variant CTX-M-199 can lead to resistance to AZA in this context, with MIC exceeding 16 mg/L [66]. In contrast, CTX-M-14 only causes a slight reduction in susceptibility, with an MIC of 4 mg/L [66]. This disparity stems from a difference in the amino acid at position 240: CTX-M-15 features glycine, whereas CTX-M-14 features aspartate [66]. In *Enterobacter mori*, the Arg244Gly mutation in SHV-12 can mediate resistance to AZA, raising the MIC from 0.5/4 mg/L to 4/4 mg/L [67]. This mutation disrupts the arginine salt bridge within the avibactam binding pocket, resulting in an increase in binding energy from −5.24 kcal/mol to −4.32 kcal/mol, as well as an increase in the inhibition constant from 143.96 μM to 677.37 μM, which indicates a decline in inhibitory efficiency of avibactam [67]. However, this mutation is also associated with reduced resistance to cephalosporins.

CMY-type AmpC is another important class of resistance gene. Specific mutations in CMY-16, such as Tyr150Ser and Asn346His, can directly reduce the effectiveness of avibactam [68]. Conversely, the synergistic interplay between CMY-42 and PBP3 insertion mutations is a more frequently observed phenomenon. CMY-42 has potent hydrolytic activity against ATM, and its presence alongside PBP3 insertions creates a solid basis for AZA resistance [69, 70]. Furthermore, bacteria can enhance their resistance levels by modulating the gene dosage of resistance determinants. For example, Inc mutations located on IncI1/IncL plasmids can increase plasmid copy numbers [71]. Alternatively, the insertion sequence IS*1294* can generate tandem repeats via rolling-circle replication or facilitate the translocation of resistance elements onto naturally high-copy, small plasmids [72]. These strategies enable exceptionally high levels of drug resistance to be mediated at a relatively minimal fitness cost.

In addition to β-lactamase and PBP3 mutations, reduced outer membrane permeability and overactive efflux pumps also contribute to AZA resistance. In *Klebsiella pneumoniae*, truncation mutations in the OmpK36 pore protein (e.g. Y310*) hinder drug entry into the periplasmic space, but reintroducing the wild-type gene restores susceptibility [65]. Concurrently, upregulation of the AcrAB-TolC efflux pump exacerbates resistance, while the addition of the PAβN inhibitor significantly reduces the MIC [62]. These findings suggest that reduced membrane permeability resulting from pore protein deficiency is a more prevalent resistance mechanism than efflux pump activation, and that the two often interact synergistically with β-lactamase mutations to form complex resistance networks.

## Clinical applications and efficacy evaluation

Two Phase III trials, REVISIT and ASSEMBLE, were conducted to evaluate the efficacy and safety of AZA in the treatment of severe infections.

The REVISIT study was a prospective, multinational, randomized, open-label but centre-blinded trial enrolling patients with cIAIs and HAP, including ventilator-associated pneumonia (VAP). Patients were randomized in a 2:1 ratio to receive AZA (1.5 g/0.5 g every 6 h, with metronidazole for cIAIs) or meropenem (1 g every 8 h) [73]. In the intention-to-treat analysis of 422 patients, the clinical cure rate was 68.4% in the AZA group and 65.7% in the meropenem group. Subgroup analysis showed similar efficacy between the two groups in cIAIs (76.4% vs 74.0%), whereas in HAP/VAP, the AZA group had a cure rate of 45.9% vs 41.7% [73]. The 28-day all-cause mortality rate was lower in the AZA group (overall 4.3% vs 7.1%; 10.8% vs 19.4% in the HAP/VAP subgroup) [73]. However, the interpretation of REVISIT for MBL-producing infections should be cautious, because only 10 patients had MBL-positive baseline pathogens, and only 7 received AZA [74].

The ASSEMBLE study specifically targeted severe infections caused by MBL-positive Gram-negative bacteria. Although the study was terminated early due to recruitment difficulties, among the 15 patients enrolled, clinical cure was observed in 42% of patients receiving AZA compared with 0% in the best-available-therapy group [75]. The 28-day mortality rate in the AZA group was lower than that in the control group (8% vs 33%) [75]. However, the very small sample size precludes any definitive conclusion regarding superiority.

Fixed-dose AZA and CZA + ATM are pharmacologically related but not identical regimens. AZA provides synchronized exposure of aztreonam and avibactam in a fixed-dose formulation, whereas CZA + ATM requires separate preparation and infusion, making administration more complex and increasing the risk of ATM-AVI exposure mismatch, even with Y-site co-infusion [61, 74]. However, current evidence does not establish categorical clinical superiority of AZA over CZA + ATM, because direct comparative trials are lacking [61, 74].

Although CZA + ATM does not effectively achieve the pharmacodynamic targets of AZA, multiple case reports have demonstrated that this regimen is effective in treating infections caused by MBL-producing *Enterobacteriaceae* (Table 4). Despite potential biases, this combination remains crucial when AZA is not readily available. The prospective observational study by Falcone et al. provides the strongest evidence: compared with other active treatments, this combination regimen significantly reduced 30-day all-cause mortality in patients with MBL-producing *Enterobacteriaceae* bloodstream infections (19.2% vs. 44%), with a hazard ratio of 0.17 in multivariate Cox regression, representing an 83% reduction in the risk of death, and a propensity score-adjusted hazard ratio of 0.37 [76]. Regarding secondary endpoints, the risk of clinical failure at 14 days was reduced by 70%, and hospital stay was significantly shortened [76]. A study by Bavaro et al. in patients with COVID-19 complicated by bloodstream infections further supports this regimen. KPC-producing strains were treated with CZA, while those producing NDM received the combination regimen [77]. Results showed similar 14-day mortality rates (26% vs. 38%) and 28-day mortality rates (35% vs. 48%) between the two groups [77]. The 14-day mortality rate was only 7% for patients who initiated appropriate treatment early, but rose to 64% when treatment was delayed beyond 72 h [77]. However, Meini et al. observed that among elderly patients with multiple comorbidities, the 30-day mortality rate remained as high as 46.7% even when all patients received this combination therapy, highlighting the significant impact of patients' baseline conditions on prognosis [78].

**Table 4.** Case reports of treatment with CZA combined with aztreonam.^152^

PubMed PMID	Number of patients receiving CZA + ATM	Sex, age	MBL-producing *Enterobacterales* pathogen(s)	Infection site	Clinical outcome
28167541	1	Female, 72 years	*Enterobacter cloacae* (NDM-1)	Skin and soft tissue	Clinical cure
30021742	1	Male, 42 years	*K. pneumoniae* (NDM-1, OXA-181, CTX-M-15)	Bone	Clinical cure
30530600	1	Female, 3 years	*Morganella morganii* (NDM-1, CMY-4)	Bloodstream	Clinical cure
30282120	1	Mae, 61 years	*E. cloacae* (NDM-1)	Skin and soft tissue	Clinical cure
31704216	1	Female, 67 years	*K. pneumoniae* (NDM-1)	Bloodstream	Clinical cure of index infection, urinary tract infection with same organism at day 30
	1	Female, 44 years	*K. pneumoniae* (NDM-1)	Respiratory	Clinical cure of index infection, respiratory with same organism at day 20
31802119	1	Male, 4 years	*Enterobacter hormaechei* (KPC-4, NDM-1)	Bloodstream	Clinical cure
33217119	1	Male, 73 years	*E. cloacae* (IMP-4)	Bloodstream	Clinical cure
	1	Male, 50 years	*E. cloacae* (IMP-4)	Bone	Clinical cure
	1	Male, 64 years	*E. cloacae* (IMP-4)	Bloodstream	Clinical cure of index infection, pyelonephritis due to the same organism at 4 months
	1	Male, 49 years	*E. cloacae* (IMP-4)	Skin and soft tissue	Clinical cure
33924769	1	Male, 57 years	*K. pneumoniae* (NDM-1)	Bloodstream	Clinical cure
33958683	1	Female, 40 years	*K. pneumoniae* (NDM-1)	Endocarditis	Clinical cure
34888479	1	Female, 45 years	*E. cloacae* (IMP-4)	Bloodstream	Clinical cure
34716170	1	Female, 30 years	*K. pneumoniae* (VIM)	Bone	Clinical cure
36225496	1	Male, 39 years	*Citrobacter sedlakii* (NDM)	Bone	Clinical cure
36371600	1	Male, 62 years	*K. pneumoniae* (NDM)	Bloodstream	Clinical cure
37601796	1	Female, 70 years	*K. pneumoniae* (NDM)	Bone	Clinical cure
	1	Male, 71 years	*E. cloacae* (NDM, OXA-48)	Bone	Clinical cure
38376188	1	Male, 30 years	*K. pneumoniae* (VIM), *Serratia marcescens* (VIM)	Bloodstream	Clinical cure
	1	Male, 30 years	*K. pneumoniae* (VIM).	Skin and soft tissue	Clinical cure
	1	Male, 81 years	*S. marcescens* (VIM)	Skin and soft tissue	Clinical cure of index infection and bacteremia from S. marcescens at day 21
	1	Male, 19 years	*S. marcescens* (VIM), *K. pneumoniae*	Skin and soft tissue	Clinical cure
38646598	1	Female, 33 years	*E. cloacae, E. coli*	Urinary tract	Clinical cure
38289725	1	Male, 12 years	*E. coli* (NDM-1)	Bloodstream	Clinical cure of index infection, bacteremia from *E. coli* at day 36 leading to death
38885998	1	Male, 40 years	*K. pneumoniae* (MBL producer)	Central nervous system	Clinical cure
39050862	1	Male, 70 years	*K. pneumoniae* (NDM, OXA-48)	Skin and soft tissue infection	Clinical cure
39494754	1	Male, 50 years	*E. coli* (NDM, OXA-48), *M. morganii*	Biliary tract infection	Clinical cure

In this context, therapeutic drug monitoring (TDM) is particularly critical for this temporary combination regimen. Clinical case reports have demonstrated that TDM can identify insufficient exposure and guide individualized dose adjustments. For example, TDM successfully helped achieve bactericidal targets in a child with bloodstream infection caused by an *Enterobacter* sp. carrying KPC and NDM [79]. In a cystic fibrosis patient, TDM revealed inadequate target attainment with intermittent infusion, and only after switching to continuous infusion was 100% *ƒ*T > MIC achieved [80]. Therefore, although CZA + ATM may be used as a temporary option when AZA is unavailable, TDM and synergy testing should be performed.

Overall, AZA is an effective treatment option for infections caused by MBL-producing *Enterobacterales.* Its mechanistic rationale, synchronized PK/PD exposure, standardized susceptibility testing, and supporting prospective Phase III data make it a practical preferred option when available [61]. CZA + ATM remains a clinically relevant and mature alternative, especially when AZA is unavailable, with encouraging observational outcomes. However, this regimen requires more complex administration, may be affected by AZA exposure mismatch, and lacks standardized susceptibility testing [61]. Therefore, in the absence of definitive clinical evidence demonstrating the superiority of AZA, regimen selection should move beyond a binary “equivalent versus non-equivalent” framework and consider drug availability, laboratory capacity, organ function, administration feasibility, pathogen species, resistance mechanisms, drug exposure, timing of therapy, age, comorbidities, and infection severity.

## Safety assessment

In the REVISIT trial, 412 patients received at least one dose of the study drug, with 275 receiving AZA and 137 receiving meropenem [73]. The incidence of treatment-related adverse events of any grade was 64% in both groups, while the incidence of serious adverse events was 19% and 18%, respectively [73]. No treatment-related serious adverse events were reported in the AZA group, whereas one case of abnormal liver function was reported in the meropenem group [73].

Common laboratory abnormalities associated with AZA include anaemia (7%), elevated ALT (7%), elevated AST (5%) and diarrhea (6%) [73]. Other events occurring in at least 3% of patients include hypokalemia (6%), fever (5%), abnormal liver function tests (4%), nausea (4%) and vomiting (4%) [73]. Hypersensitivity or allergic reactions occurred in 13% of patients, with no cases classified as severe [73]. Liver dysfunction occurred in 18% of patients, with no cases meeting the Hays criteria. And *Clostridioides difficile* diarrhea occurred in 8% of patients (4% in the meropenem group) [73].

In addition, AZA may cause uncommon reactions such as eosinophilia, leukocytosis, dizziness, hypotension and rash [81]. Rare, serious adverse reactions include pancytopenia, neutropenia, seizures, hepatitis and severe skin reactions [82]. In patients with renal impairment, reduced drug clearance may increase the risk of neurological events if the dose is too high [82]. Overall, AZA is well tolerated and has a favourable benefit-risk profile.

## Summary and outlook

AZA is currently the only BLBLIs effective against *Enterobacterales* producing MBLs. Although European and American guidelines recommend cefiderocol, which is not yet available in China and is already facing a problem of resistance [83, 84]. Therefore, AZA holds greater promise.

Two pharmacoeconomic studies published in 2025 demonstrated its superior cost-effectiveness. Compared to meropenem combined with colistin, although AZA has a higher pharmaceutical cost, the model estimated a higher clinical cure rate, lower mortality, shorter hospital stays, and reduced risk of nephrotoxicity. Consequently, it was associated with a per-patient savings of €534 in cIAIs, while in HAP and VAP, the incremental cost-effectiveness ratio is €1,552 per quality-adjusted life year, below the willingness-to-pay threshold [85]. A Spanish dynamic transmission model predicted that introducing this drug as a first-line treatment could prevent nearly 20,000 infections, save over 4,600 lives, and reduce healthcare costs by around €40.5 million over 10 years, resulting in a net monetary benefit of €1.22 billion [86]. However, these findings are derived from country-specific economic analyses and model assumptions, and their generalizability to other healthcare systems remains uncertain. Despite its high procurement cost, AZA may represent a cost-effective strategic option in settings where model assumptions, local epidemiology, drug pricing, and healthcare costs are comparable.

In summary, AZA is a valuable addition to the therapeutic arsenal for treating severe, multidrug-resistant *Enterobacterales* infections in adults, particularly in regions where MBLs are prevalent. CZA + ATM remains an important and clinically relevant alternative, particularly in settings where AZA is not available. To prevent resistance from emerging, precise prescribing guided by rapid molecular diagnostics is essential. While current resistance rates remain low, continuous surveillance is warranted. Given the suboptimal *in vitro* activity against *P. aeruginosa*, combination therapy represents a promising avenue for future development. Furthermore, additional clinical data regarding pediatric patients and specific patient populations is required.
